# Supporting emergency departments workers well-being: a systematic review of the literature on interventions

**DOI:** 10.1007/s11739-025-04225-6

**Published:** 2025-12-15

**Authors:** Benedetta Colaiacovo, Elisa Suardi, Marica Ceruti, Chiara Corvino, Luca P. Vecchio, Mara Gorli, Francesca Gianni, Barbara Muzzulini

**Affiliations:** 1 Department of Psychology, Catholic University of the Sacred Heart, Milan, Italy; 2 Department of Clinical Sciences and Community Health, University of Milan, Milan, Italy; 3 Department of Psychology, University of Milan - Bicocca, Milan, Italy; 4 Emergency Department, IRCCS Cà Granda Ospedale Maggiore Policlinico, Milan, Italy

**Keywords:** Emergency department, Healthcare workers, Well-being, Burnout, Intervention, Mindfulness, Resilience, Systematic review

## Abstract

Emergency departments (EDs) are high-pressure environments where healthcare workers face ongoing acute and chronic stressors, increasing the risk of burnout, anxiety, and depression. These challenges affect staff well-being, job satisfaction, and patient care. Although various interventions have been developed to improve well-being, their effectiveness remains insufficiently understood. This systematic review aims to examine interventions targeting the well-being of ED healthcare workers, focusing on individual, group, and organizational-level strategies. Following the PRISMA guidelines, a comprehensive search was conducted across four databases (PubMed, PsycINFO, Scopus, Web of Science) to identify peer-reviewed studies published between 2021 and 2024. A total of 26 articles met inclusion criteria, each evaluating well-being interventions for ED staff. Studies were categorized by intervention type—individual, group, organizational, or multilevel—and outcomes assessed. Most interventions targeted the individual level (n = 21), including mindfulness training, resilience programs, and educational approaches. Fewer studies addressed group (n = 1), organizational (n = 2), or multilevel (n = 2) interventions. Eighteen studies reported improvements in at least one well-being outcome, most commonly reductions in stress and burnout. However, only six included long-term follow-up. Overall, interventions in ED settings primarily focus on individual-level strategies and demonstrate short-term benefits. Less frequent multilevel approaches may provide more sustainable improvements. Future research should emphasize longer follow-up periods, robust study designs, and context-specific implementation to better assess and enhance the effectiveness of well-being interventions for ED healthcare workers.

## Introduction

Emergency departments (EDs) are widely acknowledged as high-stress work environments. In contrast to other healthcare settings, EDs are marked by unpredictable patient volumes and varying case severities, the urgency of medical interventions, and the need to address a wide spectrum of acute health conditions. These characteristics create intense pressure for healthcare professionals working in these settings [1]. Staff in EDs face continual exposure to both acute and chronic stressors, which can significantly impact their well-being [2]. Several key factors contribute to the elevated stress levels among ED personnel. These include frequent exposure to workplace violence, stringent time constraints, the emotional toll of patient care, and insufficient access to resources, whether social, physical, or psychological [3]. Additionally, the nature of ED work often involves irregular shifts that disrupt normal societal schedules, amplifying the strain on workers and contributing to high turnover rates. In turn, this turnover increases the burden on remaining staff, perpetuating a cycle of stress and overwork [4]. Together, these stressors deeply affect the overall well-being of ED healthcare professionals.

Most existing research on ED staff has centered on identifying individual and organizational stressors that negatively influence their health and performance. Burnout is one of the most frequently examined outcomes [5, 6], with reported prevalence rates among ED nurses and physicians ranging from 26 to 82% [7]. Other studies reveal that 85% of ED nurses experience at least one symptom of secondary traumatic stress [8], alongside moderate to severe anxiety [9] and moderate to high levels of compassion fatigue [10]. Furthermore, research has investigated how workplace conditions influence staff intentions to leave their jobs [4, 11].

Research on the well-being of ED workers underscores the urgent need for targeted interventions designed to improve working conditions and enhance staff well-being. As highlighted by Swancott et al. [5], such interventions can be implemented across multiple levels, namely, the individual, team, and organizational levels.

At the individual level, interventions typically focus on enhancing personal coping skills and resilience through methods such as training programs and mindfulness practices. Team-level interventions aim to strengthen interpersonal relationships and team dynamics by encouraging peer support and collaboration within workgroups [12]. At the organizational level, strategies are directed toward systemic improvements, these may include optimizing workflows, adjusting staffing models, redesigning physical spaces, or introducing tools that enhance operational efficiency.

Several literature reviews have sought to classify and assess the effectiveness of these interventions. For example, Xu et al. [51] analyzed 14 studies focusing on efforts to reduce occupational stress and burnout among ED staff. Their review identified two main categories of intervention: individual-focused approaches, such as stress management training [13], compassion fatigue resilience programs [12], and mindfulness techniques; and organizational approaches, including shift restructuring [14], interprofessional team meetings [15], and improved communication pathways with leadership [16]. Likewise, Gerrard et al. [50], in their systematic review, outlined a variety of strategies ranging from mindfulness-based programs, including tactile massage and hypnosis-based relaxation [17, 18], to video debriefing sessions and workplace wellness initiatives, such as the ED Healthy Workplace Initiative [19] and the Happiness Practice [20].

Building on these contributions, the present systematic literature review aims to expand upon previous findings and deepen the understanding of interventions that support healthcare worker well-being in EDs. This review seeks to contribute to the development of strategies that enhance both the working conditions and overall quality of life for professionals operating in these demanding environments.

## Methods

This systematic review was conducted to identify, analyze, and synthesize empirical studies evaluating interventions aimed at improving the well-being or enhancing the quality of working life of healthcare professionals working in EDs. The review followed the Preferred Reporting Items for Systematic Reviews and Meta-Analyses (PRISMA) guidelines.

### Search strategy

A systematic search was conducted across four major e-databases: PubMed, Scopus, Web of Science and PsycINFO. The search was undertaken between September and December 2024. The search strategy was designed to capture relevant empirical studies involving ED healthcare workers and interventions related to psychological well-being or quality of working life.

Keywords used to perform the search in the online databases were.

(Physician OR Physicians OR “healthcare workers” OR nurse* OR doctor OR doctors OR "medical staff") AND ("emergency room" OR "emergency medicine" OR "accident and emergency" OR "emergency department" OR "emergency ward") AND (intervention OR curriculum OR train OR program OR debrief OR "psychosocial support" OR counsel OR therapy) AND ("compassion fatigue" OR stress OR burnout OR "moral distress" OR anxiety OR depression OR "well-being" OR wellbeing OR wellness OR "job satisfaction" OR "turnover intention" OR "work-related quality of life").

### Inclusion criteria

Studies were included if they met the following criteria:

1. Studies published in English or Italian in peer-reviewed journals from December 2021 onward were included. Previous systematic reviews by Xu et al. [51] and Gerrard et al. [50] covered literature up to November 2021; therefore, the current review builds upon their work by including studies published from that point forward.
2. Studies that included a clear intervention component specifically targeting ED healthcare workers' well-being or work-related quality of life.
3. Studies that focused on clinical healthcare staff (e.g., nurses, physicians, allied health professionals) working specifically in ED settings, regardless of educational background or years of experience.

### Exclusion criteria

Studies were excluded based on the following conditions:

1. Articles not published in English or Italian, or not undergone a peer-reviewed publication process (e.g., conference abstracts, theses, or grey literature).
2. Articles that did not include an implemented intervention (e.g., protocols, theoretical models, or reviews), or in which the intervention was not directly focused on staff well-being or quality of working life in EDs.
3. Studies that did not focus on permanent clinical staff working in emergency departments (e.g., studies involving administrative personnel, patients, medical students, residents, or healthcare workers from other hospital departments).
4. Studies that did not include any post-intervention measurement to assess the effectiveness of the intervention.

### Study selection

**Fig. 1:**
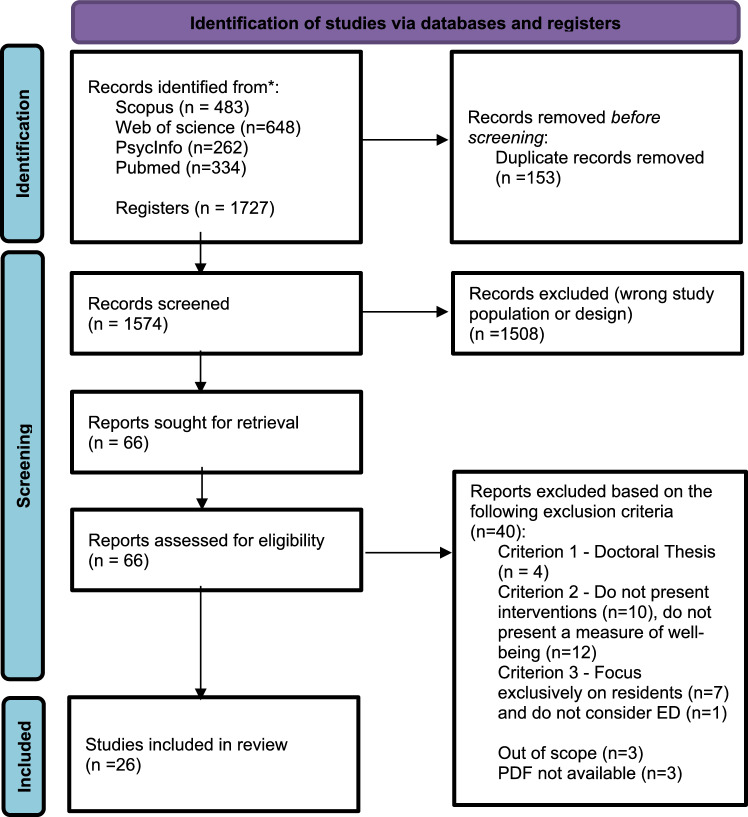
PRISMA 2020 flow diagram (From: Page MJ, McKenzie JE, Bossuyt PM, Boutron I, Hoffmann TC, Mulrow CD, et al. The PRISMA 2020 statement: an updated guideline for reporting systematic reviews. BMJ 2021; 372: n71. https://doi.org/10.1136/bmj. n71.)

All identified records were imported into RefWorks (ProQuest), where duplicates were removed. Titles and abstracts were initially screened against the eligibility criteria. Full texts of potentially relevant articles were then retrieved and assessed independently by four reviewers (BM, BC, ES, MC). In cases of disagreement, articles were retained if at least three out of four reviewers agreed on their inclusion, ensuring decisions were based on substantial consensus. Reasons for excluding full-text articles were documented and are presented in the PRISMA flow diagram (Fig. 1).

### Data extraction

Data were independently extracted by the same four reviewers using a standardized template. Extracted variables included:

- Year of publication
- Country of study
- Study design (e.g., quasi-experimental, randomized controlled trial)
- Type and content of the intervention
- Methodology and tools used
- Target population characteristics
- Intervention duration
- Use of a control or comparison group
- Measures of well-being or quality of working life
- Reported effectiveness
- Follow-up period (if applicable) (i.e. number of post-test)

Included studies were also categorized based on the level at which the intervention was implemented, following a four-tier framework: individual-level, group-level, organizational-level, and multilevel interventions.

- Individual-level interventions focused on enhancing personal coping resources and skills. These typically involved educational programs on resilience and communication, mindfulness-based practices, cognitive-behavioral strategies, and stress-reduction techniques.
- Group-level interventions leveraged the group context to promote well-being, often through formats such as focus groups, peer support networks, and team-based activities aimed at fostering interpersonal support and collaboration.
- Organizational-level interventions targeted systemic aspects of the ED work environment. Strategies in this category included modifications to resource allocation, physical infrastructure, job design, workload distribution, and shift scheduling.
- Multilevel interventions combined elements across two or more levels, such as integrating individual training with organizational changes, to produce a more comprehensive and sustained impact on staff well-being.

Intervention outcomes were evaluated based on the classification by Schneider et al. [15], which includes positive well-being indicators (e.g., job satisfaction, work engagement), affective symptoms and negative psychological outcomes (e.g., emotional exhaustion, PTSD symptoms), cognitive-behavioral variables (e.g., turnover intention, organizational commitment, role behaviors) and physical health complaints (e.g., somatic symptoms, fatigue).

### Risk of bias

The methodological quality of the included interventional studies was assessed using the Downs and Black checklist. A total of 22 studies were evaluated, excluding those with an observational design. Of these, seven were randomized controlled trials (RCTs), while the remaining employed quasi-experimental designs, including pre-post studies with control groups or post-test-only formats. The RCTs scored between 84 and 88% (27–28 out of 32), indicating a low risk of bias. Non-randomized studies showed more variation, with scores ranging from 50 to 81% (16–26 out of 32), reflecting a broader spectrum of methodological quality. Overall, 59.1% of studies (13 out of 22) were classified as high quality (≥ 23 points), while 36.4% (8 studies) were considered of moderate quality (17–22 points). Only one study (4.5%) was identified as having a high risk of bias, with a score of 16 or below. Lower-scoring studies often lacked random allocation, baseline (pre-intervention) measurements, and proper adjustment for confounding variables, along with incomplete reporting of adverse events and recruitment strategies. Conversely, several quasi-experimental studies achieved high methodological scores by including control groups, providing clear intervention protocols, and applying thorough statistical analyses, even without randomization. These findings suggest that, although RCTs offer the highest internal validity, a substantial portion of non-randomized interventional studies included in this review also demonstrated strong methodological rigor and reliability.

## Results

The search process is summarized in the PRISMA flow diagram (Fig. 1). A total of 1,727 records were identified through database searches. After removing duplicates, 1,574 articles were screened by title and abstract. Of these, 1,508 were excluded for not meeting the inclusion criteria, primarily due to ineligible populations or study designs. After full-text assessment, 26 studies published between December 2021 and 2024 were included in the final analysis. The substantial heterogeneity in study designs, methodology, participant characteristics, intervention types, and outcome measures made direct comparisons difficult and precluded the possibility of conducting a meta-analysis.

### Study characteristics

The included studies were primarily conducted in the United States (n = 5), Iran (n = 5), Canada (n = 3), and the Netherlands (n = 2). Sample sizes ranged from 14 to 578 participants. Twelve studies focused exclusively on nurses, six on broader ED staff (including administrative and allied health professionals), four on physicians alone, and four included both nurses and medical staff with two also including residents. In terms of study design, seventeen studies were classified as quasi-experimental, meaning they involved an active intervention without random allocation of participants. These included designs such as one-group pre-post evaluations and post-test-only formats, reflecting pragmatic approaches to studying real-world healthcare settings. Seven studies were randomized controlled trials (RCTs), which involve the random assignment of participants to intervention or control groups and are considered the gold standard for minimizing bias. One study used a mixed-methods approach, integrating both qualitative and quantitative data to examine outcomes from multiple perspectives. The dominance of quasi-experimental designs highlights the practical and ethical challenges of conducting randomized trials in emergency department environments. Nonetheless, several of these studies demonstrated strong methodological quality through careful design, use of control groups, and comprehensive reporting of outcomes, supporting their value within the evidence base despite the lack of randomization.

All studies used self-reported psychometric instruments to measure outcomes. Burnout was the most frequently assessed variable (n = 9), often in combination with job satisfaction [21, 22], stress [23, 24], anxiety [25], work engagement [26], or turnover intention [27]. Stress was measured in seven studies, one of which also included physiological parameters [28]. Other outcomes included anxiety and depression [30, 31], moral sensitivity and caring behaviors [31], and subjective evaluations of the intervention [33, 34]. Overall, 18 studies reported significant improvements in well-being outcomes at post-intervention (T2). However, only six studies included a follow-up phase to assess long-term effects (T3).

### Intervention types

The review identified a variety of interventions classified according to the level at which they operate, following the framework proposed by Xu et al. [51]. Organizational-directed interventions aim to modify organizational factors or introduce policy and procedural changes to improve the working environment. Examples cited by Xu et al. [51] include adjustments to shift schedules and/or reductions in working hours or workloads. Individual-focused interventions are designed to strengthen personal coping abilities or stress-management skills. These interventions target the single healthcare professional and include strategies such as mindfulness programs or psychological training. Group-level interventions focus on teams or work units, promoting practices such as peer support or structured debriefings. Finally, multilevel interventions integrate components across individual, group, and/or organizational domains, aiming for a more systemic and comprehensive impact on staff well-being (Table 1).

**Table 1:** Characteristics of the studies included in the review

Authors	Country	Population	N population	Control group (Yes/No)	Type of intervention	Measures of well-being	Duration	Study design	N post-test (s)	Intervention details	Short term (Yes/No)
*Educational interventions*											
Arbuzia et al. [34]	Iran	Nurses	N = 60	Y	Resilience training	Stress	2 months	Quasi-experimental study	1	Eight 30-min sessions of resilience training	N
Bagherzadeh et al. [31]	Iran	Nurses	N = 140	Y	Skill training	Moral sensitivity, caring behaviours	3 weeks	Quasi-experimental study	2	Six 60-min training sessions	Y (but not at T3)
Dorscheidt et al. [35]	Netherlands	MD and residents	N = 58	Y	Resilience training	Stress and resilience	8 weeks	Randomized controlled trial	1	Online training program through weekly emails	N
Hasani et al. [37]	Iran	Nurses	N = 54	Y	Skill training	Stress	2 months	Randomized controlled trial	2	Eight sessions of 45-mintraining classes twice a week	Y (also at T3)
Hines-Stellisch et al. [27]	USA	Nurses	N = 10	N	Wellness coaching	Burnout and turnover intention	6 months	Quasi-experimental study	1	Eight online modules within weekly sessions of 30–60 min	Y
Khan et al. [21]	Pakistan	MD, nurses (residents)	N = 200	Y	Aggression management discussion	Burnout, de-escalation of violence, overall satisfaction, fear of violence and coping with patients’ aggressions	6 months	Quasi-experimental study	1	Participants received computer-based training, simulation training on de-escalation and restraint application	N
Liao et al. [30]	China	Nurses	N = 71	Y	Comprehensive Active Resilience Education (CARE)	Connor—Davidson Resilience Scale—10 (CD-RISC-10); HADS (anxiety and depression); Cognitive Emotion Regulation Questionnaire; organizational support; trait coping style		Quasi-experimental study	1	Eight modules each with bi-weekly offline session	Y
Masa'deh et al. [38]	Jordan	MD and nurses	N = 330	N	Aggression management program	Stress	3 months	Quasi-experimental study	1	‘‘LOWLINE’’ method (Lowry, Lingard, &Neal, 2016)	Y
Menchetti and Pham [39]	Canada	MD	N = 60	N	Mentorship program	Career development, academic promotion, emotional wellbeing, and niche development	6 months (o 1 year?)	Quasi-experimental – post-test only	1	Mentorship programme	Y
Monfries et al. [36]	Canada	All staff	N = 20	Y	Resilience training	Burnout, mindfulness, resilience	3 months	Randomized controlled trial	1	"Headversity™ (headversity.com) smartphone application	Y
Rose et al. [40]	Canada	All staff	N = 30	N	Debriefing workshop	Stress, wellbeing and impact of debriefings on their clinical practice	7 months	Quasi-experimental- post-test only	1	INFO debriefing training and individual interviews	Y
Shopen et al. [22]	Israel	MD	N = 20	N	Burnout reduction workshop	Burnout and job satisfaction	4 months	Quasi -experimental study	2	Group and individual discussions	N (also at T3)
*Mindfulness-type- intervention*											
Argyriadis et al. [32]	Greece	Nurses	N = 14	Y	Mindfulness	Systolic and diastolic blood pressure; heartbeats; state of sleep	1.5 weeks	Randomized controlled trial	1	Mindfullness meditation practices through online application	Y
Ito-Masui et al. [41]	Japan	MD and nurses	N = 61	N	CBT—Cognitive Behavioural Theory	Total sleep time and subjective well-being	4 weeks	Randomized controlled trial	1	Internet-delivered CBT (Cognitive Behavioural Therapy) through a smartphone app. Wrist-worn fitness tracker 24 h a day and daily questionnaires about well-being and activities	Y
Kadhim et al. [44]	Iran	Nurses	N = 80	Y	Mindfulness-Based Stress Reduction (MBSR)	Fatigue and job satisfaction	8 weeks	Quasi-experimental study	1	Eight mindfulness training sessions	Y
Lycke et al. [33]	Sweden	Nurses	N = 51	N	Mindfulness-Based Stress Reduction (MBSR)	Compassion, empathy, stress	8 weeks	Quasi-experimental study	1	Three hours introductory workshop, 8-week mindfulness-based stress reduction (MBSR) program and follow-up workshop three weeks afterward	Y
Mäkinen et al. [23]	Finland	All staff	N = 170	N	Mindfulness-Based Stress Reduction (MBSR) and Mindfulness-Based Cognitive Therapy (MBCT)	Burnout, stress, work satisfaction	3 months	Quasi-experimental study	1	Daily individual mindfulness training exercises through a smartphone app and six two hours in-class group meetings led by an instructor	Y
Pascual et al. [42]	USA	MD, nurses (residents)	N = 32	Y	Mindfulness-Based Stress Reduction (MBSR)	Heart-rate variability (HRV), a biomarker for relaxation, anxiety	4 weeks	Randomized Controlled trial	1	Guided meditations sessions via a VR headset or smartphone application during worktime	N
Pratt et al. [25]	USA	Nurses	N = 102	Y	Smartphone app-guided mindfulness practice	Burnout, distress, anxiety and depression	4 weeks	Quasi-experimental study	1	Self-directed mindfulness program through smartphone application with daily reminders asking participants to complete their mindfulness sessions and surveys when appropriate	Y
Wong et al. [43]	USA	All staff	T1 = 75 T2 = 69	N	On-shift mindfulness	Burnout	3 months	Quasi- experimental study	1	Mindfulness, breathing exercises, meditation, yoga/stretching, and relaxation techniques during work shifts	N
Xu et al. [24]	Australia	All staff	N = 148	Y	Smartphone app-guided mindfulness practice	Burnout, mindfulness, well-being	4 weeks	Randomized controlled trial	2	Daily 10 min guided mindfulness sessions through smartphone application	Y (also at T3)
*Group-level interventions*											
Connors et al. [29]	USA	MD	N = 24	N	Peer support group sessions	Burnout, distress, anxiety and depression	8 weeks	Quasi-experimental study	1	Eight online peer support group sessions once a week	N
*Organizational-level interventions*											
Calamassi et al. [28]	Italy	Nurses	N = 54	Y	Music therapy	Level of state anxiety and vital parameters	2 months	Observational study	1	Listening to music during the work break	Y
Fattahpour et al. [46]	Iran	All staff	N = 52	N	Music therapy	Stress, Productivity, satisfaction	3 months	Quasi-experimental pre-post study	1	Music (nature sounds) exposure for 60 min at the beginning and end of each work shift	Y
*Multi-level interventions*											
De Wijn et al. [26]*	Netherlands	Nurses	T1 = 578 T2 = 511 T3 = 533	N	Psychosocial safety climate (PSC)	Burnout and work engagement	2.5 years	Mixed-methods study	2	Semi-structured interviews, ‘inspiration sessions’, advice report to managers, and implementation of Psychosocial safety climate (PSC) interventions through organizational changes	Y (also at T3)
Yang et al. [45]	Taiwan	Nurses	T1 = 169 T2 = 174 T3 = 173	N	Group brainstorming strategies	Stress	2 months	Quasi-experimental study	2	Focus group discussions followed by two cycles of improvement strategies with 30-days interval	Y (also at T3)

The column “N post-test(s)” indicates the number of post-intervention assessment points included in each study. The column “Short term (Yes/No)” refers to whether the intervention demonstrated short-term effectiveness (Y = yes, N = no), defined as statistically significant improvements at the first post-intervention assessment (T2). In case the intervention demonstrate long-term effectiveness is indicated as (also at T3).The follow-up assessments is reported in the same column the time frame covered by T3 measurements and to indicate whether effects were sustained beyond the short ter

Most studies included in this review (n = 21) implemented individual-level interventions, one study employed a group-level approach, two focused on organizational-level interventions, and two adopted multilevel strategies.

### Individual-level interventions

Among the 21 studies using individual-level approaches, interventions were classified as either educational (n = 12) or mindfulness-based (n = 9).

#### Educational interventions

Educational interventions varied in content, delivery, and duration. Five studies focused on resilience training [31, 35–38]. In particular, in Hasani et al. [38], a resilience training program was delivered through eight 45-min sessions held twice weekly, aiming to reduce stress among nurses. Similarly, in the study by Arbuzia et al. [34], the intervention group received eight 30-min sessions of resilience training over two months, while the control group received no intervention. Two other studies focused on aggression management training [22, 39]. In particular, the quasi-experimental study by Masa'deh et al. [38] introduced both verbal and non-verbal communication techniques designed to help healthcare professionals de-escalate aggressive behavior. One study examined skill training focused on enhancing moral sensitivity and caring behaviors [31]. Another implemented a coaching program aimed at reducing burnout and turnover intentions [27], while a further study evaluated a mentorship initiative addressing career development, academic advancement, emotional well-being, and professional growth for physicians [39]. Additional interventions included a debriefing workshop [40] and a burnout reduction program [22]. The duration of interventions ranged from 3 weeks [31] to 7 months [40], with the majority (n = 9) lasting between 2 and 6 months. All studies included post-intervention outcome assessments, and three incorporated follow-up evaluations at a third time point (T3) [23, 32, 38]. Seven studies reported significant improvements in well-being outcomes—such as moral sensitivity, stress, and burnout—following the intervention. However, among the studies with follow-up, only one [37] demonstrated sustained long-term effects, whereas in another [22], the initial reduction in burnout was not maintained at the 3-month follow-up.

#### Mindfulness-type interventions

Eight studies focused on mindfulness-type interventions, while one employed an app-based cognitive behavioral therapy (CBT) program. The CBT intervention provided personalized sleep advice from specialists and encouraged self-reflection using data from fitness trackers and daily well-being questionnaires [41]. The mindfulness-type-type interventions varied considerably in terms of intensity, delivery methods, and duration across studies. Some were app-based [25, 26], while others used virtual reality (VR)–guided meditations accessed via a VR headset, or a standalone mobile app located in the emergency department (ED) on-call room [42]. Several interventions incorporated mindfulness practices in combination with breathing exercises, meditation, yoga/stretching, and relaxation techniques, which were designed to be practiced during work shifts and included all ED staff, including administrative personnel [43]. In terms of duration, four studies implemented the intervention over four weeks, two lasted three months, one was conducted over eight weeks, and another was nearly one weeklong. Most studies (n = 7) reported positive effects on healthcare workers’ well-being, primarily through reductions in burnout and stress, as well as improvements in sleep quality (n = 5). One study also assessed anxiety levels alongside burnout following the intervention [25], while another reported increased job satisfaction and reduced fatigue [44]. Additionally, one study monitored physiological changes such as heart-rate variability (HRV) and a relaxation biomarker, which were measured during each mindfulness session [42]. Two studies explored participants' perceptions of the intervention using semi-structured interviews, thus assessing effectiveness qualitatively rather than quantitatively [33, 34]. One study reported no significant change in outcome scores pre- and post-intervention. Notably, most studies assessed outcomes immediately after the intervention. Only four included a follow-up at a later time point (T2), and one study extended the evaluation to a third time point (T3), three months post-intervention [24].

### Group-level interventions

One study adopted a group-level intervention [29]. In this case, the group setting was used as a vehicle for promoting change. Specifically, Connors et al. [29] conducted a pilot study to assess the feasibility, receptivity, and preliminary effectiveness of peer support groups in addressing anxiety, depression, distress, and burnout among ED physicians. The intervention consisted of eight weekly online peer support sessions. Each session began with a brief 2- to 3-min check-in, allowing participants to share their current experiences. This was followed by a group discussion focused on common and urgent issues and concluded with the sharing of positive action plans or inspirational reflections. The groups were facilitated by a non-clinician peer support leader and one of three trained emergency medicine physician co-facilitators.

### Organizational-level interventions

Two studies implemented organizational-level interventions [29, 47], both involving modifications to the physical work environment. Specifically, these interventions exposed staff to various types of music or natural sounds during work breaks or shifts. For instance, in the study by Fattahpour et al. [46], a music player was used for 60 min at the beginning and end of each shift, playing nature sounds, such as a blend of bird songs and waterfall audio—selected from a dedicated sound database. Calamassi et al. [28] examined the effects of listening to music during work breaks, comparing it to routine breaks without music, in reducing stress and anxiety levels among ED nurses.

Although these interventions did not target traditional organizational aspects such as workload, work shifts, or task structure, they introduced a change in the physical work environment—namely, the presence of different types of music during shifts or breaks. Therefore, these interventions directly influenced the physical work context in which ED’s staff operate and thus we classified them as organizational-level interventions.

### Multilevel interventions

This review identified two studies that implemented multilevel interventions [27, 46]. In the study by De Wijn et al. [26], a multilevel intervention was developed based on a comprehensive risk assessment and targeted both individual and organizational levels. The process began with a well-being survey and semi-structured interviews to identify key risks and gather contextual insights. This was followed by inspiration sessions to build knowledge, culminating in a management report with tailored recommendations. A Psychosocial Safety Climate (PSC) intervention was subsequently implemented. Organizational strategies included increasing the number of emergency department (ED) nurse trainees and support staff, scheduling medical specialists during peak hours, reorganizing patient flow by creating low- and high-care units, and enhancing security measures, such as installing staff-only access doors. At the individual level, the intervention introduced psychoeducation on burnout, coaching to enhance team communication, work shift adjustments to allow for rest, and the implementation of self-rostering to increase scheduling autonomy. The intervention spanned 2.5 years and incorporated both pre-post measures and follow-up assessments. In terms of outcomes, most job-related factors improved, with the exception of autonomy, which showed only a temporary increase. Notably, work engagement declined, and burnout levels remained stable.

The second study, conducted by Yang et al. [45]], implemented a multilevel intervention during the COVID-19 pandemic, using an action research approach. The process involved online surveys and group brainstorming to assess stress levels among ED nurses. The survey, based on a COVID-19-specific stress questionnaire, examined the sources and intensity of stress as well as nurses’ needs. Intervention strategies, developed over two iterative cycles, addressed three main areas: (1) infection protection (e.g., ultraviolet disinfection, personal protective equipment, health screenings), (2) workload reduction (e.g., training for junior nurses, shift limitation, increased staffing), and (3) body-mind-social well-being (e.g., meal delivery, bonus incentives, improved communication, and a more supportive work environment) [45].It is important to note that this intervention was implemented in the specific context of the COVID-19 pandemic, and its strategies may not be easily transferable to other settings or periods. Nevertheless, multilevel interventions, though limited in number, tend to be supported by more robust research designs and reflect a greater emphasis on contextual adaptation and implementation processes. First, thorough preliminary assessments help ensure that interventions are tailored to the specific needs of participants. Second, these interventions often extend over longer periods, such as in De Wijn et al. [26], allowing more time for stakeholder engagement and transparent communication, which can reduce resistance to change and improve implementation success.

## Discussion

### Summary of the finding

This systematic review analyzed 26 studies published between December 2021 and 2024 that focused on interventions designed to improve the well-being of healthcare professionals, particularly those working in emergency departments (EDs). The studies encompassed a wide range of designs, populations, intervention types, and outcome measures, resulting in considerable variability across the evidence base. Regarding study design, 7 studies adopted a randomized controlled trial (RCT) design, 17 used a quasi-experimental design, and 1 employed a mixed-methods approach. Among the reviewed studies, 21 implemented individual-level interventions. These primarily targeted personal resilience, mindfulness, or psychological well-being. Educational and mindfulness-based approaches were the most commonly employed intervention types. Group-level (n = 1), organizational-level (n = 2), and multilevel (n = 2) interventions were less frequently represented in the literature. Organizational-level interventions targeting the well-being of ED staff often aim to address structural and environmental stressors. These include: (1) improving occupational safety and wellness by involving staff in decision-making and improving communication with management [47] (2) addressing workplace stressors via structured, multi-disciplinary meetings to co-develop practical solutions [15]; (3) reducing physician burnout via telemedicine shifts that enabled senior physicians to work remotely on weekends [14]; and (4) improving team coordination and operational efficiency by implementing the Training Within Industry (TWI) management model to eliminate unnecessary processes and streamline ED workflows [48]. In contrast, only two studies in our review adopted an organizational-level intervention, both focusing on modifying the physical work environment using music and sound exposure [29, 47]. Eighteen studies reported statistically significant improvements in well-being at the immediate post-intervention time point (T2). Reported outcomes included reductions in burnout, stress, and anxiety, along with improvements in job satisfaction and sleep quality. Six studies included follow-up assessments at a third time point (T3), with mixed findings regarding the sustainability of intervention effects. A small number of studies incorporated physiological markers such as heart rate variability or qualitative data collection to assess outcomes. Intervention duration varied widely: educational programs ranged from three weeks to seven months, and mindfulness-based interventions from one week to three months. Most studies were conducted in high-resource settings, including the USA, Iran, Canada, and the Netherlands. Participants were predominantly nurses, with fewer studies including physicians, allied health professionals, or administrative staff.

### Key trends emerging from the review

The results of this review highlight several consistent patterns in the existing evidence base. First, the predominance of individual-level interventions was evident across the included studies, with most focusing on approaches such as resilience training, mindfulness-based stress reduction, or other psychological skills programs. These interventions frequently reported short-term improvements in well-being and related outcomes at immediate post-intervention assessments. However, only six studies included follow-up measurements, and their results were mixed, providing limited information on whether benefits persist over time. This pattern indicates that the current literature offers substantially more evidence on short-term effects than on long-term impact.

A second observable trend is the scarcity of organizational and multilevel interventions. Only two studies combined individual- and organizational-level components, while organizational-level approaches alone were also rare. This distribution aligns with what has been described in previous reviews, such as Xu et al. [51], which similarly noted a predominance of individual-level strategies. In this review, the limited number of organizational or systemic interventions restricts the available evidence on how changes in workplace structures, processes, or management practices may influence staff well-being. Although several external studies have highlighted the relevance of organizational factors, these findings were referenced only to contextualize the observed scarcity of such interventions in the current body of research.

Within the two multilevel studies identified, interventions involved actions across different levels of the emergency department system and included broad staff engagement. However, because these studies represent a small portion of the total evidence base, conclusions about their effectiveness remain tentative. Their presence nonetheless illustrates that more complex, system-wide approaches have been implemented, albeit infrequently.

A further methodological pattern emerging from the review concerns the timing of outcome assessment. The majority of studies evaluated effects either immediately post-intervention or within a short period thereafter. Long-term follow-up was uncommon, which substantially limits the ability to determine the durability of observed effects. The current evidence therefore does not allow clear differentiation between interventions that produce sustained changes and those associated primarily with short-term improvements.

Overall, the findings of the review indicate that most available evidence pertains to individual-level interventions and short-term outcomes, with comparatively limited data on organizational, multilevel, and long-term effects.

### Limitations and future directions

This review presents several limitations that should be considered when interpreting its findings. First, there was substantial heterogeneity across the included studies in terms of design, methodology, participant characteristics, intervention type, and outcome measures. This variability limited direct comparability and precluded the possibility of conducting a meta-analysis, making it difficult to identify consistent patterns across interventions.

Most studies relied on self-reported psychometric tools to assess outcomes. Although these instruments are widely used, they remain susceptible to reporting biases and may not fully capture the multidimensional nature of well-being. Only a small number of studies incorporated additional objective or qualitative measures, indicating a methodological gap. Future research could benefit from integrating mixed-methods designs to provide a more comprehensive evaluation of intervention effects.

The duration, intensity, and scope of interventions varied widely, from very brief programs to multi-month initiatives. The predominance of short-term interventions, combined with the limited number of follow-up assessments (n = 6), restricts conclusions regarding the durability of effects. Future studies should therefore include longer follow-up periods and multiple time-point assessments to better understand whether improvements are sustained over time.

Geographical distribution represents another limitation. Most studies were conducted in high-resource settings such as the USA, Iran, Canada, and the Netherlands, raising questions about generalizability to lower-resource environments or health systems with different structures. Similarly, the predominance of nurses in study samples limits the applicability of findings across the wider emergency department workforce, including physicians, allied health professionals, and administrative staff. Future research should adopt sampling strategies that reflect the diversity of ED roles and expand to a wider range of geographic and socioeconomic contexts.

A further observation from this review is the predominance of individual-level interventions, which generally demonstrated short-term improvements. Organizational and multilevel interventions were less common, limiting the available evidence on approaches that operate across broader system levels. While previous literature has highlighted the importance of organizational conditions in shaping workplace well-being, in this review such elements appeared infrequently within intervention designs. Future studies could explore multilevel frameworks more thoroughly, as well as investigate how structural and procedural aspects of ED environments—such as staffing patterns, workflow organization, communication, and team dynamics—interact with individual-level strategies.

Finally, the review focused exclusively on emergency department personnel. This choice reflects the unique clinical and organizational characteristics of ED settings, but also limits the applicability of findings to other hospital contexts. Thus, we encourage future reviews to consider examining interventions implemented in other clinical units to identify potentially transferable elements that could inform ED-specific approaches.

## Conclusion

This systematic review shows that most existing interventions aimed at improving the well-being of emergency department healthcare workers operate at the individual level and generally report short-term improvements. The review also identifies a relative scarcity of organizational and multilevel interventions, resulting in limited data on approaches that address broader workplace structures or combine individual and systemic components. Overall, the current evidence base provides a clearer understanding of short-term effects than of sustained or system-wide impacts, indicating areas where further research is needed.
